# Separation of glycine-rich proteins from sea hare eggs and their anti-cancer activity against U937 leukemia cell line

**DOI:** 10.17179/excli2016-293

**Published:** 2016-06-01

**Authors:** Won Woo Lee, Won-Suck Kim, Ginnae Ahn, Kil-Nam Kim, Soo-Jin Heo, Moonjae Cho, I. P. Shanura Fernando, Nalae Kang, You-Jin Jeon

**Affiliations:** 1 School of Marine Biomedical Sciences, Jeju National University, Jeju 63243, Republic of Korea; 2 College of Medical and Life Sciences, Silla University, Busan, 46958, Republic of Korea; 3 Department of Marine Bio-Food Sciences, Chonnam National University, Yeosu 59626, Republic of Korea; 4 Jeju center, Korea Basic Science Institute (KBSI), Jeju 690-140, Republic of Korea; 5 Global Bioresources Research Center, Korea Institute of Ocean Science & Technology, Jeju, Republic of Korea; 6 Department of Biochemistry, College of Medicine, Cheju National University, Jeju 63349, Republic of Korea

**Keywords:** sea hare egg (SE), glycine-rich protein, anti-cancer effect, apoptosis, mitochondrial pathway

## Abstract

The present study was designed to investigate the anti-cancer effects of *Sea hare* eggs (SE) in U937 cells and its major active components. The aqueous extract of SE (ASE), which contained the highest protein content, dose-dependently inhibited the cancer cell's growth (IC_50_ value, 10.42 ± 0.5 µg/mL). Additionally, ASE markedly caused DNA damage by inducing apoptotic body formation, DNA fragmentation, and accumulation of sub-G_1_ DNA contents. ASE induced apoptosis by activating caspase-3 and 9 and poly (ADP-ribose) polymerase (PARP) by regulating the expression of Bcl-2/Bax. Moreover, among its molecular weight fractions, the > 30 kDa fraction showed the highest cell-growth-inhibitory effects, which was inhibited by heat treatment. Furthermore, the > 30 kDa fraction had markedly higher glycine content than the ASE. The presence of two protein bands at around 16 and 32 kDa was identified. In addition, two fractions, F1 and F2, were obtained using anion-exchange chromatography, with the F1 having an improved cell-growth-inhibitory effect than the > 30 kDa fraction. Taken together, these results suggest that the ASE contains glycine-rich proteins, including the active 16 and 32 kDa proteins, which account for its anti-cancer effects by inducing apoptosis via regulation of the mitochondrial pathway.

## Introduction

Apoptosis is one of the most important cellular mechanisms and involves a selective process of physiological cell deletion. Deregulation of this process contributes to a variety of diseases, particularly cancer (Moon et al., 2007[32]). Normally, this process occurs in two phases: the initiation phase that may be stimulus-dependent and a common downstream effector phase that involves chromatin condensation, DNA fragmentation, and alterations in the cell membrane (Decker et al., 2001[7]). In particular, members of the Bcl-2 family, including both the anti-apoptotic Bcl-2 and the pro-apoptotic Bax (Bcl-2-associated X protein) proteins, are known to regulate mitochondrial and cellular physiology (Murphy et al., 2005[33]). In addition, the regulation of mitochondrial function plays a key role in initiating apoptosis in cancer cells (Budihardjo et al., 1999[5]). Thus, it was recently suggested that cancer chemotherapeutics exert their pharmacological effects partly by triggering apoptotic cell death, making the induction of apoptosis in cancer cells a target for cancer treatment (Ahmad et al., 1997[1], Kim et al., 1999[21]). Many researchers have tried to discover or develop drugs that can regulate the function of mitochondria for the treatment of a variety of cancers (Decker et al., 2001[7]; Kim et al., 2010[20]; Zhang et al., 2012[43]; Jose and Rossignol, 2013[16]).

Sea hares belong to the species *Aplysia* of subclass Opis-(Imbrandii), including *Aplysia kurodai, Aplysia juliana, *and *Dolabella auricuralia *within the phylum Mollusca. Their yellow-colored eggs are laid in gelatinous strings during their spawning season (May and June) (Kisugi et al., 1987[22]). In previous studies, several cytotoxic proteins, aplysianin A, P, and E, had respectively been isolated from the albumen gland, purple fluid, and eggs of *A. kurodai*. Among the purified sea hare glycoproteins, aplysianin A, P, E, and dolabellanin A exhibited not only anti-tumor and antineoplastic effects, but also antibacterial activity in both Gram-positive and Gram-negative bacteria (Kisugi et al., 1987[22], 1989[23]; Iijima et al., 1994[15], 1995[14]). However, the underlying biological mechanisms of the anti-tumor and anti-neoplastic effects induced by the cytotoxic properties of sea hare eggs and its components have not yet been reported. Despite the fact that they have excellent cytotoxic effects on tumor cells, there is no reported evidence of the anti-tumor activity of sea hare eggs (SE) obtained from the coast of Jeju Island in South Korea or of their underlying biological mechanisms. Therefore, in the present study, we investigated the effects of ASE on both growth inhibition and cellular DNA damage that induce apoptosis via the mitochondrial pathway in U937 cells. We also evaluated the anti-cancer effects of the molecular weight fractions of ASE and their major amino acid composition. Additionally, we isolated the two active fractions by anion-exchange chromatography and identified their anti-cancer effects. 

## Materials and Methods

### Materials 

RPMI-1640 medium, fetal bovine serum (FBS), penicillin-streptomycin, and Dulbecco's Phosphate Buffered Saline (DPBS) were purchased from Gibco-BRL (Burlington, Ont, Canada). 3-(4,5-Dimethylthiazol-2-yl)-2,5-diphenyltetrazolium bromide (MTT), dimethyl sulfoxide (DMSO), Hoechst 33342, ribonuclease A (RNase A), and propidium iodide (PI) were obtained from Sigma (St. Louis, MO, USA). DNA ladder size markers were purchased from Invitrogen (Carlsbad, CA, USA). Antibodies against Bcl-2, Bax, caspases 3 and 9, cleaved PARP, and β-actin were purchased from Cell Signaling Technology (Bedford, Massachusetts, USA). Protein marker was purchased from Bio-Rad (Richmond, CA, USA). Caspase 3 activity assay kit was purchased from Promega (San Luis Obispo, CA, USA). All other chemicals and reagents used herein were of analytical grade. 

### Preparation of an aqueous extract from Sea hare eggs (SE) (ASE)

Sea hare eggs were collected along the coast of Jeju Island, Korea, between June 2007 and August 2008. The samples were washed thrice with tap water to remove salt, epiphytes, and other debris attached to the surface. Afterward, they were carefully rinsed with fresh water, freeze-dried, and pulverized into powder. The powdered SE (10 g) was dissolved in distilled water (500 mL) and extracted at 20° C for 24 h under continuous shaking, followed by centrifugation (3000 rpm, 20 min, 4° C) to obtain the supernatant. The aqueous extract from SE (ASE) was then lyophilized and dissolved in cell culture media for further studies. 

### Analysis of chemical composition and amino acid composition 

The proximate chemical composition including ash, moisture, proteins, carbohydrates, and lipid contents of the sample was analyzed according to the slandered methods of the Association of Official Analytical Chemists (AOAC). The amino acid composition of the samples was analyzed by an amino acid auto-analyzer (S430, SyKam, Gewerbering, German) using a Cation separation column (LCA K07/Li).

### Fractionation of ASE according to molecular weight using ultrafiltration system

Four fractions (< 1 kDa, 1-10 kDa, 10-30 kDa, and > 30 kDa) were prepared from ASE using a Millipore Laboratory-scale TFF ultrafiltration system (Millipore Corporation, Bedford, MA) with 1, 10, and 30 kDa molecular weight cut-off (MWCO) membranes. This is a known technique for isolating hydrophilic compounds (Ahn et al., 2011[2]). The amino acid composition of the > 30 kDa fraction was analyzed by an Amino Acid Analyzer. 

### Sodium dodecyl sulfate-polyacrylamide gel electrophoresis (SDS-PAGE) 

The active fraction of proteins > 30 kDa (10 μg) was subjected to electrophoresis with a 15 % SDS-PAGE, and the gel was stained using silver staining assays.

### Cell culture 

U937 (human leukemia cells), B16F10 (mouse melanoma cell line), and HeLa (woman cervical carcinoma cell line) cells were cultured using RPMI-1640 medium supplemented with 10 % (v/v) heat-inactivated FBS and 1 % (v/v) penicillin-streptomycin. The cultures were maintained at 37° C in a humidified atmosphere supplemented with 5 % CO_2_.

### MTT assay 

Effects of ASE on the cancer cells' (U937, B16F10, and HeLa) growth were assessed via a colorimetric MTT assay. The cells (2 × 10^4^ cells/well) were seeded in 96-well culture plates and incubated with various concentrations of ASE and its four fractions for 72 h. After incubation, an MTT stock solution (50 μL; 2 mg/mL in PBS) was added to each well and the cells were further incubated for 4 h. The plates were centrifuged for 10 min at 2000 rpm and the supernatants were aspirated. The formazan crystals in each well were then dissolved in DMSO (200 μL). The amount of purple color formazan was assessed by measuring the absorbance at 540 nm. 

### Alkaline comet assay 

An alkaline comet assay was used as previously described to determine the DNA damage induced by ASE in the U937 cells (Kang et al., 2007[17]). The cells (4 × 10^5 ^cells/well) were seeded into a 24-well culture plate and incubated at 37° C supplemented with 5 % CO_2_ in a humidified atmosphere with various concentrations of ASE (6.25, 12.5, and 25 µg/mL). After 72 h, the cells were collected and mounted on microscopic slides. Then the cells were lysed using lysis buffer (2.5 M NaCl, 100 mM Na_2_-EDTA, 10 mM Tris, and 1 % Triton X-100, pH 10) for 1 h at 4° C and were subjected for electrophoresis. After the electrophoresis, DNA tailing on the slides were observed under a fluorescence microscope and analyzed by the Komet 5.5 program (Kinetic Imaging, Liverpool, UK). The percentage of fluorescence in the DNA comet tail of 50 cells per slide was recorded. 

### Nuclear staining with Hoechst 33342 

The formation of apoptotic bodies in the cells was identified using the cell-permeable DNA dye Hoechst 33342. Cells with homogeneously stained nuclei were considered viable, whereas the presence of chromatin condensation and/or fragmentation were indicative of apoptosis (Gschwind and Huber, 1995[10], Lizard et al., 1995[31]). U937 cells were placed in 24-well plates at a concentration of 4 × 10^5^ cells/well. The cells were then treated with various concentrations of ASE (6.25, 12.5, and 25 µg/mL) and incubated for an additional 72 h. Hoechst 33342 was then added to the culture medium to a final concentration of 10 µg/mL, and the plates were re-incubated for an additional 10 min at 37° C. The stained cells were then observed under a fluorescence microscope equipped with a CoolSNAP-Pro color digital camera in order to determine the degree of nuclear fragmentation. 

### Determination of DNA fragmentation 

The characteristic ladder pattern of DNA breakage was analyzed via agarose gel electrophoresis. The U937 cells were plated on 6-well plates at a concentration of 4 × 10^5^ cells/well. The cells were incubated with various concentrations of ASE (6.25, 12.5, and 25 µg/mL) for 72 h. The DNA was isolated with a Promega Wizard^® ^Genomic DNA Purification Kit (Promega, Madison, WI, USA) and electrophoretically analyzed on 1.2 % agarose gel containing 0.1 µg/mL ethidium bromide.

### Propidium iodide (PI) staining assay 

Cell cycle analysis was conducted to determine the proportion of apoptotic sub-G_1_ hypodiploid cells (Nicoletti et al., 1991[35]). U937 cells were plated on 6-well plates at a concentration of 4 × 10^5 ^cells/mL. The cells were then treated with ASE (6.25, 12.5, and 25 µg/mL) and were incubated for 72 h. The cells were then harvested and fixed in 70 % ethanol (1 mL) for 30 min at 4° C. The cells were then washed twice with DPBS and incubated in darkness in 1 mL of DPBS containing 100 μg PI and 100 μg RNase A for 30 min at 37° C. A flow cytometric analysis was conducted with a FACSCalibur flow cytometer (Becton Dickinson, San Jose, CA, USA). 

### Preparation of cytosolic proteins

U937 cells (4 × 10^5^ cells/mL) were harvested for the experiment after 72 h incubation with ASE (6.25, 12.5, and 25 µg/mL). Cell lysates were prepared with a lysis buffer (50 mM Tris-HCl at pH 7.4, 150 mM NaCl, 1 % Triton X-100, 0.1 % SDS, and 1 mM EDTA). After centrifugation, the collected protein concentrations were determined using a BCA^TM^ protein assay kit.

### Western blot analysis 

The proteins (30 μg each) were subjected for SDS-PAGE and the gels were transferred onto nitrocellulose membranes (Bio-Rad, Hercules, CA, USA). The membranes were incubated with primary antibodies against Bax, Bcl-2, caspase 3, cleaved caspase 9, cleaved PARP, and β-actin in TTBS (25 mM Tris-HCl, 137 mM NaCl, and 0.1 % Tween 20, pH 7.4) containing 2 % nonfat dry milk for 1 h. The membranes were then washed with TTBS and incubated with secondary antibodies, either anti-mouse IgG or anti-rabbit IgG (Santa Cruz Biotechnology, CA, USA). The blots were developed by enhanced chemiluminescence reagents (iNtRON, Sungnam, Korea) according to the manufacturer's instructions. 

### Measurement of caspase 3 activity 

Effects of ASE on caspase 3 activity were identified using a Caspase 3 activity kit according to the manufacturer's instructions. This assay is based on the capacity of the active enzyme to cleave DEVD-pNA (Asp-Glu-Val-Asp-p-anilide), which is a known substrate. The proteins (50 μg each) and 50 µM DEVD-pNA were incubated for 16 h at 37° C. The caspase-3 activity was then measured spectrophotometrically at 405 nm.

### Anion exchange chromatography

The selected active fraction (> 30 kDa) was further purified using a DEAE-cellulose anion-exchange column. The column was initially washed with a 50 mM sodium acetate (pH 5.0) buffer followed by equilibration with 0.2 M NaCl in a 50 mM sodium acetate (pH 5.0) buffer. After sample injection, the column was washed with the same 0.2 M NaCl in 50 mM sodium acetate (pH 5.0) buffer and its ionic strength was gradually increased to 1.2 M NaCl, facilitating the separation of the sample’s components into individual components as a function of its charge.

### Statistical analysis 

Data were analyzed by one-way ANOVA (analysis of variance) and were reported as mean ± standard error (S.E.). Where appropriate, the data were compared using unpaired Student’s t-test. Results were considered to be statistically significant if p < 0.05.

## Results

### Sea hare eggs (SE) and its aqueous extract (ASE) contained higher protein and carbohydrate content

Analysis of the SE chemical composition revealed that SE and its aqueous extract (ASE) contained significantly high amounts of proteins and carbohydrates. Particularly, the protein contents of SE and ASE were 36.1 ± 0.6 % and 30.8 ± 0.3 % respectively and were higher than those of carbohydrates were (22.5 ± 0.4 % and 20.9 ± 2.8 %, respectively). Additionally, the extraction yield of ASE was 26 %. 

### Growth inhibition activity of ASE on U937 (human leukemia cell line)

**Figure 1:**
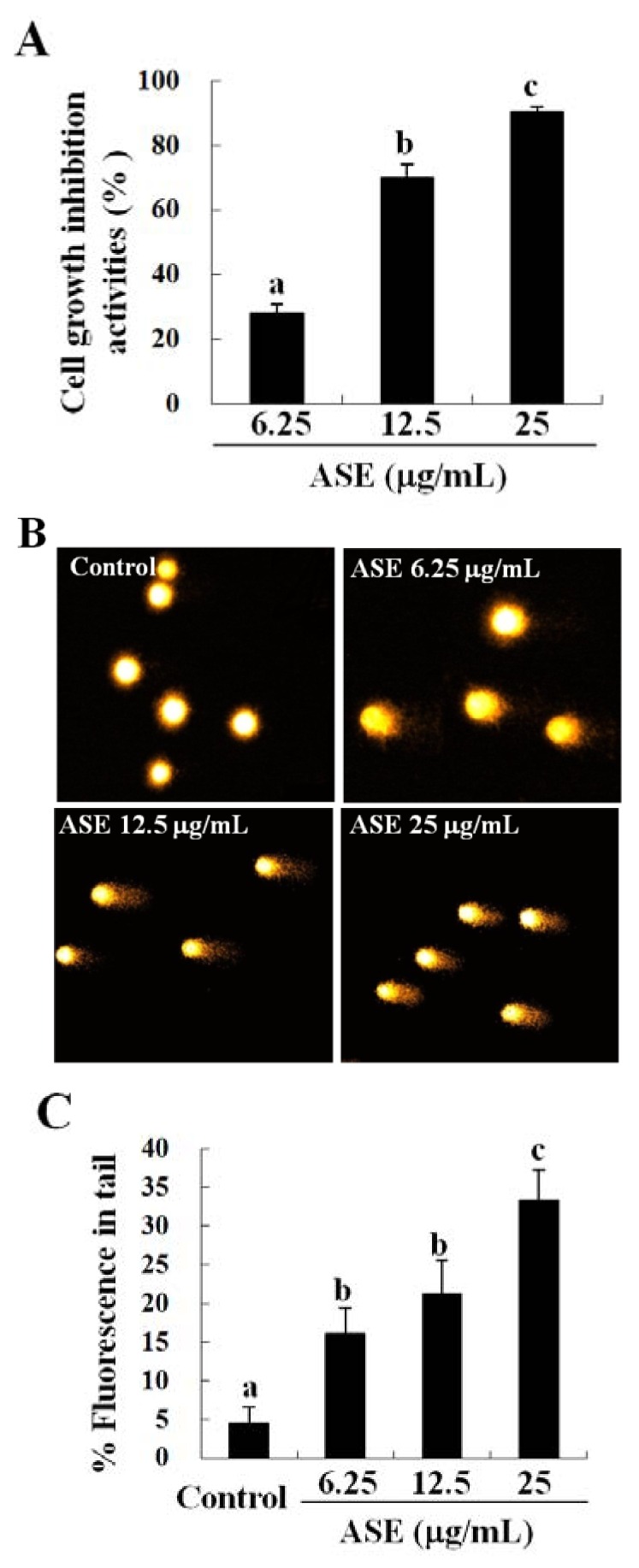
Anti-cancer activity of ASE. (A) Growth inhibition and (B) photomicrographs of DNA damages in U937 cells. The cells (2 × 10^4^ cells/well) incubated with ASE (6.25, 12.5 and 25 µg/mL) for 72 h were used for the MTT assay. (C) Percentages of tail DNA detected by alkaline comet assay. Statistical evaluation was performed to compare the experimental groups and the corresponding control groups. The results are representatives of three separate experiments. The results were considered to be statistically significant if *p* < 0.05.

Anticancer activities of ASE were investigated by an MTT assay. Growth inhibitory effects were evaluated using U937, B16F10, and HeLa carcinoma cell lines and the results demonstrated that ASE markedly inhibited the growth of all cancer cells (Table 1(Tab. 1)). The growth inhibitory effect of ASE was remarkably higher in U937 cells (IC_50_ values: 10.4 ± 0.5 µg/mL), than in the other cell lines in which the IC_50_ values were 29.6 ± 0.7 µg/mL and 22.2 ± 1.2 µg/mL respectively for the B16F10 and the HeLa cells. Hence, the U937 cell line was selected for further experimentation. Additionally, within the 6.25 µg/mL and 25 µg/mL concentration ranges, ASE significantly inhibited the growth of U937 cells in a concentration-dependent manner (Figure 1A(Fig. 1)). 

### ASE increased cellular DNA damage in U937 cells in a dose-dependent manner

An alkaline comet assay was used to determine whether the growth-inhibitory effects of ASE on U397 cells were associated with cellular DNA damage. As indicated in Figure 1B(Fig. 1), ASE dose-dependently increased the percentage of tail DNA, indicating a pronounced DNA damage, in U937 cells within ASE concentrations of 6.25 μg/mL and 25 μg/mL, than was observed in the untreated control cells. In particular, the 25 µg/mL ASE concentration induced a higher DNA damage (33.37 ± 2.02 %) than was observed in the untreated control cells (4.58 ± 3.86 %) (Figure 1C(Fig. 1)). 

### ASE elevated the formation of apoptotic bodies and DNA fragmentation in nuclei of U937 cells

**Figure 2:**
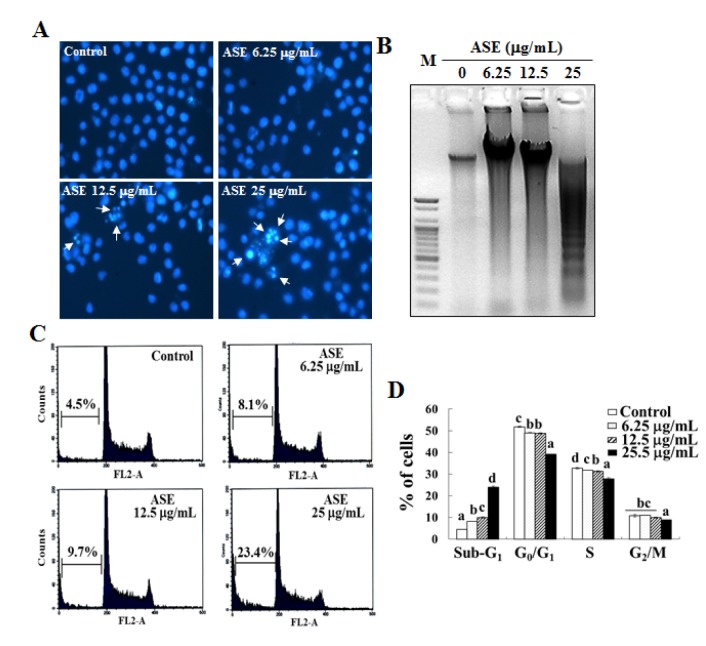
Induction of apoptosis by ASE in U937 cells. (A) Apoptotic body formation and (B) DNA fragmentation in U937 cells. (C and D) DNA contents of sub-G1 phase in U937 cells. The cells (4 × 10^5^ cells/mL) were incubated with ASE (6.25, 12.5 and 25 µg/mL) for 72 h. Apoptotic bodies were stained with Hoechst 33342 solution and then observed under a fluorescent microscope. Fragmented DNA was extracted and analyzed on 1.5 % agarose gel containing EtBr. The results are representative of three separate experiments. Cell-cycle analysis was done with PI staining method and analyzed via flow cytometry. The results are representatives of three separate experiments. The results were considered to be statistically significant if *p* < 0.05.

Further experiments were performed to evaluate ASE-induced formation of apoptotic bodies and fragmentation of DNA that signify apoptosis in nuclei of U937 cells. The use of fluorescence-based microscopy with a cell-permeable DNA dye Hoechst 33342, is generally considered the most reliable method to identify apoptotic cells. The microscopic images demonstrated that thecells of the untreated control group (Figure 2A(Fig. 2)) had intact nuclei, whereas the ASE-treated cells showed an increased nuclear fragmentation and the formation of apoptotic bodies. This dramatic increase in nuclear fragmentation was observed to be dose-dependent within 6.25 µg/mL and 25 µg/mL of the ASE concentration range.

Thus, agarose gel electrophoresis was used to analyze the effects of ASE, which can lead to DNA fragmentation in U937 cells (Figure 2B(Fig. 2)). As indicated in Figure 2B(Fig. 2), DNA fragmentation was not induced in the untreated control cells, whereas ASE (from 6.25 µg/mL to 25 µg/mL) led to an increase in DNA fragmentation in a dose-dependent manner. Particularly, the 25 µg/mL ASE concentration showed a clear increase in DNA ladders, a phenomenon of DNA fragmentation that was absent in the untreated control cells. 

### ASE dose-dependently increased apoptotic sub-G_1_ DNA content in U937 cells

**Figure 3:**
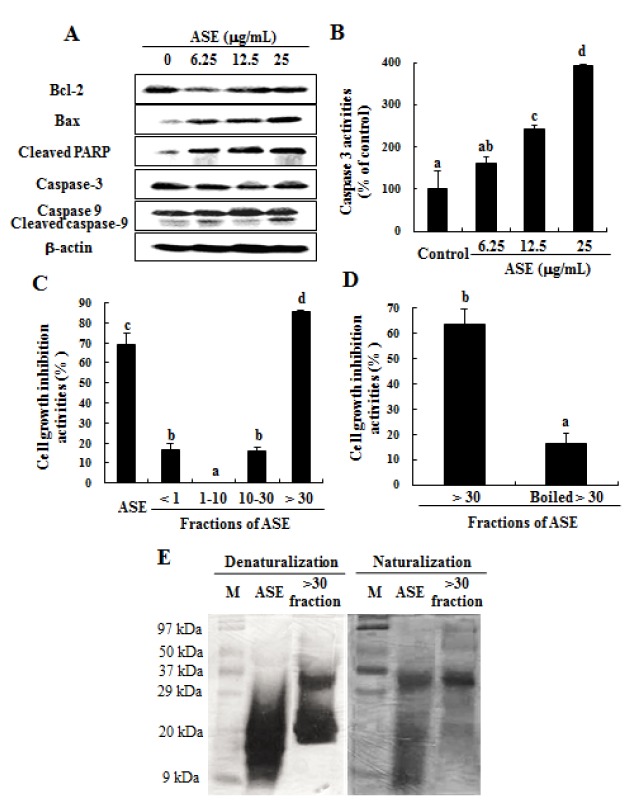
Regulation of apoptosis in U937 cells. (A) Effect of ASE on the expression of apoptosis-related proteins and (B) the activation of caspase 3 in U937 cells. (C) Effect of four molecular fractions from ASE and their boiled > 30 kDa fraction on the growth inhibition of U937 cells. (D) Effect of > 30 kDa fraction and the boiled > 30 kDa fraction (12.5 µg/mL) for 72 h on the growth inhibition of U937 cells. (E) SDS-PAGE analysis of cell lysates. Equal amounts of cell lysates (30 μg) were resolved via SDS-PAGE and stained by silver staining. The results are representatives of three separate experiments. The results were considered to be statistically significant if *p* < 0.05.

A propidium iodide (PI) staining assay and subsequent cell cycle analysis were performed to illustrate the relationship between ASE-induced apoptosis and the cell cycle phases. As indicated in Figures 2C and D(Fig. 2), analysis of DNA content in the untreated control cells revealed a nearly 4.5 ± 0.1 % apoptotic sub-G_1_ DNA content. However, there was a significant accumulation of cells with sub-G_1_ DNA content in a concentration-dependent manner after 72 h of ASE treatment (Figure 3(Fig. 3)). Treatment with 25 µg/mL of ASE caused a marked increase in sub-G_1_ DNA content to nearly 24.0 ± 0.6 % (Figure 3D(Fig. 3)), which was about 5 times higher than in the untreated control cells. 

### ASE decreased the expression of anti-apoptotic molecules and increased pro-apoptotic molecules in U937 cells

Western blot analysis was performed to assess the effect of ASE on the expression of apoptotic molecules. As shown in Figure 3A(Fig. 3), ASE down-regulated the expression of Bcl-2 at all concentrations (from 6.25 µl/mL to 25 µl/mL) than that observed in the untreated control cells. However, the expression of Bax was markedly up-regulated in the presence of ASE, whereas the expression of BCl-2 was down-regulated than those in the untreated control cells. In addition, ASE effectively activated caspase 3 and 9, inducing their cleavage at all concentrations (Figure 3A(Fig. 3)). Moreover, PARP cleavages were markedly increased by treatment with ASE at all concentrations than that observed in the untreated control cells (Figure 3A(Fig. 3)). Further analysis showed that ASE significantly increased caspase 3 activity in a dose-dependent manner than that observed in the untreated control cells (Figure 3B(Fig. 3)). 

### The > 30 kDa fraction of ASE produced the highest growth inhibitory effect against U937 cells and was markedly decreased by the inactivation of its protein content

Four different ASE fractions were initially prepared in accordance with their molecular sizes using an ultra-filtration system. The recovery rate of proteins in ASE and its > 30 kDa fraction was 14.0 % and 7.1 % respectively. The MTT assay demonstrated that the > 30 kDa fraction had the highest growth-inhibitory effect on U937 cells (Figure 3C(Fig. 3)). However, this effect was markedly inhibited via inactivation of the > 30 kDa fraction by heat treatment (Figure 3D(Fig. 3)), indicating that inactivation of the protein components in the > 30 kDa fraction by heat treatment caused a decline in its cell-growth-inhibitory effects. Furthermore, the SDS-PAGE analysis of the cell lysates indicated that the heat treatment caused a substantial change in the protein distribution pattern of the ASE- and > 30 kDa-treated cells, indicating denaturalization of its proteins (Figure 3E(Fig. 3)). This further explains the cause for the similar results observed in the cell growth inhibition assays.

### The high molecular weight > 30 kDa fraction consisted of high glycine contents and could be the key anticancer component of proteins against U937 cells

As demonstrated in Table 2(Tab. 2), the > 30 kDa fraction contained higher amounts of glycine (30.9 ± 0.0 %), isoleucine (11.9 ± 0.1 %), valine (11.5 ± 0.1 %), and tyrosine (10.4 ± 0.1 %) than those of the ASE, with the highest anti-cancer effect on U937 cells than the ASE and the other fractions (< 1 kDa, 1-10 kDa, and 10-30 kDa fractions). In addition, the major protein bands in the > 30 kDa fraction of ASE were clustered at 15-20 kDa and 30~35 kDa sizes.

### Further separation of > 30 kDa fraction and the growth inhibitory effects of the separated fractions on U937 cells

**Figure 4:**
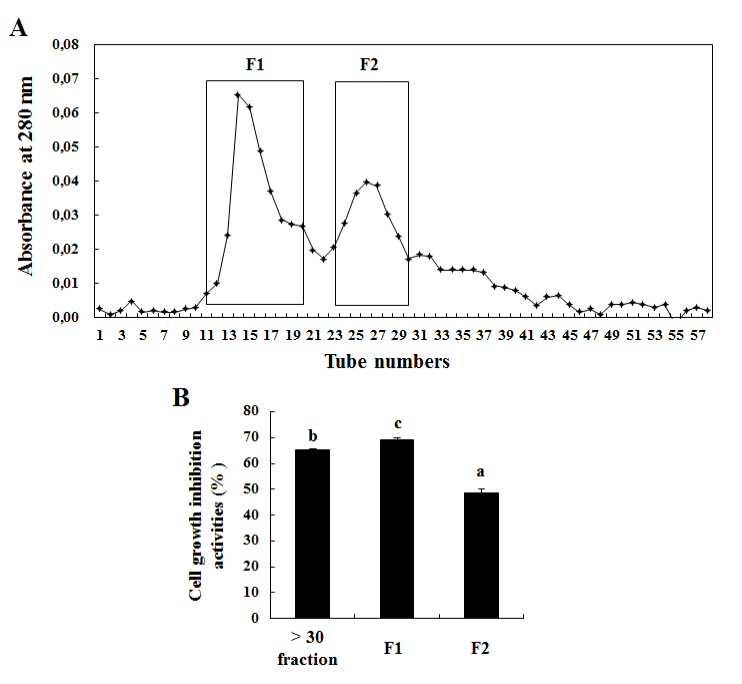
Chromatogram of active components purified from > 30 kDa fraction by anion-exchange chromatography (A) and its cell-growth-inhibitory effect (B). (A) The > 30 kDa fraction was analyzed using anion-exchange chromatography and two fractions were purified. (B) The cells (2 × 10^4^ cells/well) incubated with > 30 kDa fraction and its purified two fractions for 72 h were used for the MTT assay. The results are representatives of three separate experiments. The results were considered to be statistically significant if *p* < 0.05.

Anion-exchange chromatography was successfully used to further separate the > 30 kDa fraction into 58 sub-fractions. Two fractions, F1 and F2, were purified from the > 30 kDa fraction (Figure 4A(Fig. 4)) following the evaluation of their growth inhibitory effects on U937 cells. According to Figure 4B(Fig. 4), the > 30 kDa fraction and its two subfractions (F1 and F2) showed the highest growth inhibitory effect on U937 cells after 24 h; particularly, F1 showed the highest growth inhibitory effect on U937 cells. These two bands could be representing the two protein bands observed by Kawsar et al. (2011[19]) at 16 and 32 kDa. 

## Discussion

The present study revealed the effects of ASE on growth inhibition and cellular DNA damage in U937 cells, as well as the induction of apoptosis via the mitochondrial pathway. The > 30 kDa fraction of ASE, which accounted for the highest anti-cancer effect, consisted of glycine-rich proteins. Additionally, two active fractions were purified by anion-exchange chromatography and their anti-cancer effects were subsequently evaluated. Our results suggest that ASE consists of a higher amount of proteins that inhibited the growth of U937 cells by damaging their cellular DNA at a high rate. ASE also elevated the formation of apoptotic bodies and DNA fragmentation in nuclei of U937 cells. DNA fragmentation is known to be a biochemical hallmark of apoptosis and was discovered in nuclear DNA extracted from apoptotic cells. These results further indicate that U937 cells underwent apoptosis after ASE treatment, suggesting a correlation between the extent of apoptosis and the inhibition of cell growth. During apoptosis, DNA of individual cells appears as a hypodiploid sub-G_0/1_-peak because of partial DNA loss (Ehemann et al., 2003[8]). Indeed, ASE induced apoptosis by increasing the apoptotic sub-G_1_ DNA content of U937 cells. Collectively, these results suggest that ASE reduced the growth of U937 cells by increasing their sub-G_1_ DNA content, as well as forming apoptotic bodies and tail DNA.

Apoptosis is normally initiated through two distinct pathways: the mitochondrial pathway, and the death-receptor pathway (Budihardjo et al., 1999[5]). The Bcl-2 family, which includes both anti-apoptotic (Bcl-2 and Bcl-xL) and pro-apoptotic (Bax and Bad) proteins, affects these two pathways. Previous studies have indicated that Bax is essential for death-receptor-mediated apoptosis in cancer cells (LeBlanc et al., 2002[26]), whereas the carboxyl-terminal of Bcl-2-cleavage products trigger cell death (Cheng et al., 1997[6]). Additionally, the Bcl-2 protein, a suppressor of programmed cell death, homodimerizes and forms heterodimers with a homologous protein, Bax, which is a promoter of cell death (Hanada et al., 1995[12]). Thus, the expression of apoptosis-related proteins, including Bcl-2 and Bax, helps to elucidate the mechanism of ASE and its relation to the apoptotic process in U397 cells at 72 h. According to the current evaluation, the decreased Bcl-2 expression and the increased Bax expression induced by ASE play a key role in the apoptosis of U937 cells. Additionally, these indicate that ASE regulation of Bcl-2 and Bax proteins affects the mitochondrial and death receptor pathways. Further experiments were undertaken to test the hypothesis that ASE was associated with other apoptotic regulatory proteins such as PARP or caspase 3 and 9, all of which are included in the aforementioned pathways. Generally, the decreased expression of Bcl-2 and the increased expression of Bax, promote the expression of cytochrome C and procaspase 9 (Kluck et al., 1997[24]; Bossy-Wetzel and Green, 1999[4]). In addition, activated caspase 9 can cleave and activate downstream effector caspases such as caspase 3, 6 and 7 (Guerrero et al., 2008[11]). As reported in previous studies, activation of caspase 3 occurs during several different forms of apoptoses and has been proposed to be a crucial step in initiating apoptosis (Lazebnik et al., 1994[25]; Nicholson et al., 1995[34]; Schlegel et al., 1996[38]). Poly (ADP-ribose) polymerase (PARP) is an important nuclear enzyme, participating in DNA repair and genome surveillance; its specific cleavage has been shown to be a common marker of caspase-3 activation at the onset of apoptosis (Tanaka et al., 1984[39]; Kaufmann et al., 1993[18]; Lazebnik et al., 1994[25]). In accordance with the latter findings, ASE-induced PARP cleavage led to the activation of caspases 3 and 9, which directly or indirectly induced the formation of apoptotic bodies in the nuclei and caused DNA fragmentation, as well as increased the DNA contents in the sub-G_1_ phase in U937 cells. In addition, the proteolytic cleavage of PARP in response to caspase 3 may function to conserve cellular energy required for the apoptotic process (Wyllie et al., 1980[41]). This indicates that ASE can induce apoptosis in U937 cells via cellular energy generated from the proteolytic cleavage of PARP and caspase 3. 

Interestingly, among the molecular weight fractions of ASE, the > 30 kDa fraction had the highest growth-inhibitory effect in U937 cells. In addition, the > 30 kDa fraction had markedly higher glycine contents than ASE. These results could suggest that the plentiful glycine components in the > 30 kDa fraction play an important role in its anti-cancer effects in U937 cells. A major portion of sea hare eggs is the protein component, which has antineoplastic activity (Yamazaki et al., 1985[42]). Glycine- or proline-rich glycoproteins isolated from *Solanum nigrum Linne* and *black soybean***,** glycine itself and glycine- or proline-rich peptides have been reported to express anticancer activity in colon and colorectal carcinoma, leukemia and breast cancer cells (Liao et al., 2001[30]; Lee et al., 2004[29]; Heo and Lim, 2005[13]; Lee et al., 2005[27]; Oh and Lim, 2007[36]; Lee and Lim, 2008[28]; Okoko and Awhin, 2010[37]). In addition, previous studies have indicated that glycoproteins that contain more than 50 % hydrophobic amino acids, including glycine and proline, have these components playing a key role in its beneficial anti-cancer effects (Lee et al., 2004[29]; Heo and Lim, 2005[13]; Lee et al., 2005[27]; Oh and Lim, 2007[36]; Lee and Lim, 2008[28]). Interestingly, a recent study indicated that the molecular mass of a polypeptide purified from SE by gel permeation chromato-graphy or lactosyl-agarose affinity chromatography appeared to be 32 and 16 kDa under non-reducing and reducing conditions respectively (Kawsar et al., 2011[19]). Our findings suggest that the major components of the > 30 kDa fraction might be approximately 32 kDa and 16 kDa in size, with high amounts of glycine. In addition, the application of the ultra-filtration system in the present study, for preparing the > 30 kDa fraction was extremely successful, similar to the application of gel permeation chromatography or lactosyl-agarose affinity chromatography in the purification of polypeptides. Findings revealed that the glycine-rich protein component of the > 30 kDa fraction of ASE is a key component that confers an anti-cancer effect by inducing cellular damages via regulation of apoptosis in U937 cells. Furthermore, the two sub-fractions (F1 and F2) purified by anion-exchange chromatography, particularly the F1 fraction, showed the highest cell-growth-inhibitory effect in U937 cells. These results suggest that the two fractions could be representing the two protein bands observed by Kawsar et al., (2011[19]) at 16 and 32 kDa.

In addition, previous reports have also demonstrated that the glycine- and proline-rich glycoproteins, which consists of carbohydrates (69.74 %) and proteins (30.26 %), can stimulate mitochondria-mediated apoptotic signaling (cytochrome *c*, caspase 3, and PARP) and inhibit the activities of NF-κB in hepatocellular carcinoma cells (Oh and Lim, 2007[36]). The inhibition of NF-κB activity is closely related to its anti-cancer, anti-resistance, and apoptosis activities in various cancer cells, such as hepatocellular carcinoma and leukemic cancer cells (Foo and Nolan, 1999[9]; Arsura and Cavin, 2005[3]; Wang et al., 2010[40]). Thus, our data indicate that ASE and its active components might produce their anti-cancer effects by increasing apoptosis via inhibiting NF-κB activation in U937 cells.

## Conclusion

The present study revealed that a glycine-rich protein fraction purified from ASE exhibits anti-cancer activity by increasing apoptosis via regulation of the mitochondrial pathway in U937 cells. Although attempts were made to further purify the selected fraction, further studies are needed to evaluate the effects of the purified ASE proteins on the NF-κB pathway during apoptosis, as well as to isolate and sequence the amino acids in the specific peptides/proteins that are responsible for the observed anti-cancer effects of ASE. 

## Notes

WonWoo Lee and Won-Suck Kim contributed equally to this study.

## Acknowledgement

This research was financially supported by the Ministry of Education (MOE) and the National Research Foundation of Korea (NRF) through the Human Resource Training Project for Regional Innovation (NRF-2012H1B8A2025863). 

## Conflict of interest

The authors declare that they have no conflict of interest.

## Figures and Tables

**Table 1:**
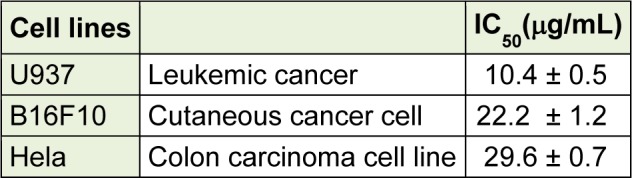
IC_50 _values of ASE on growth of B16F10, U937, and Hela cells

**Table 2:**
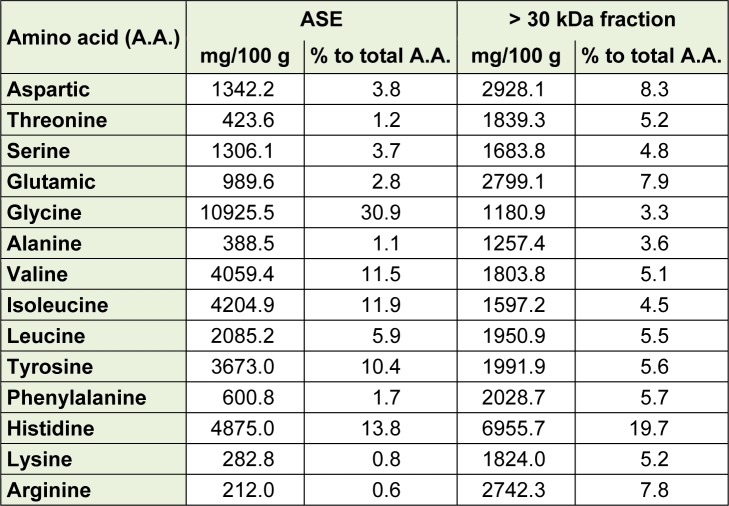
Amino acid composition in the > 30 kDa fraction of ASE
